# Supplementary material for: p53 in AgRP neurons is required for protection against diet-induced obesity via JNK1
Source: Nat Commun. 2018 Aug 24;9:3432. doi: 10.1038/s41467-018-05711-6 (PMC6109113; doi:10.1038/s41467-018-05711-6)
Supplement: Supplementary file 1 — Supplementary information [file 41467_2018_5711_MOESM1_ESM.pdf]

Supplementary table 1. Antibody used for western blot and immunohistochemistry determination.

Peptide/protein target	Name of Antibody	Manufacturer, catalog	Species raised in; monoclonal or polyclonal	Dilution used
Acetil-p53	Acetyl-p53 (Lys 379) Rabbit Antibody	Cell Signaling, #2570	rabbit, polyclonal	1:500
ATF6α	Anti-ATF-6α antibody	Santa cruz biotechnology, sc-22799	rabbit, polyclonal	1:1000
BAX	Bax Antibody	Cell Signaling, #2772	rabbit, polyclonal	1:1000
Caspase 3	Caspase-3 (8G10)Rabbit mAb	Cell Signaling, #9665	rabbit, monoclonal	1:1000
Caspase 7	Caspase-7 Antibody	Cell Signaling, #9492	rabbit, polyclonal	1:1000
CHOP	Anti-CHOP (GADD R-20) Antibody	Santa cruz biotechnology, sc-793	rabbit, polyclonal	1:1000
GAPDH	Anti GAPDH Mouse mAb (6C5)	Millipore, CB1001	mouse, monoclonal	1:5000
GRP78	Anti GRP78 antibody	Cell Signaling, #3183	rabbit, polyclonal	1:1000
IL-1β	Anti-IL1 beta antibody	Abcam, ab9722	rabbit, polyclonal	1:500
IL-6	Anti-IL6 antibody	Abcam, ab6672	rabbit, polyclonal	1:500
IRE 1	Anti IRE 1 antibody	Abcam, ab37073	rabbit, polyclonal	1:1000
JNK1/3	Anti JNK (c-17)antibody	Santa cruz biotechnology, sc-474	rabbit, polyclonal	1:1000
MKK4	SEK1/MKK4 Antibody	Cell Signaling, #9152	rabbit, polyclonal	1:1000
MKK7	MKK7 Antibody	Cell Signaling, #4172	rabbit, polyclonal	1:1000

**p53**	**p53 (1C12) Mouse mAb**	**Cell Signaling, #2524**	**mouse, monoclonal**	**1:1000**
**P66-shc**	**Anti-SHC (phosphoS36) antibody**	**Abcam, ab54518**	**mouse, monoclonal**	**1:1000**
**PARP**	**PARP Antibody**	**Cell Signalling, #9542**	**rabbit, polyclonal**	**1:1000**
**phospho-AMPKα**	**Anti phospho-AMPKalfa (Thr172)(40H9) Antibody**	**Cell Signalling, #2535**	**rabbit, monoclonal**	**1:1000**
**phospho-JNK**	**Anti-SAPK/JNK (Thr183/Tyr185) antibody**	**Cell Signalling#4668**	**rabbit, monoclonal**	**1:1000**
**phospho-p53**	**Phospho-p53 (Ser15) Antibody**	**Cell Signaling, #9284**	**rabbit, polyclonal**	**1:500**
**phospho-PERK**	**Anti-phospho PERK(Thr 981)**	**Santa cruz biotechnology, sc-32577**	**rabbit, polyclonal**	**1:1000**
**UCP1**	**Anti-UCP1 antibody**	**Abcam, ab10983**	**rabbit, polyclonal**	**1:10000**
**XBP-1**	**Anti XBP1 antibody**	**Santa cruz biotechnology, sc-7160**	**rabbit, monoclonal**	**1:1000**
**α-tubulin**	**Anti-α-tubulin antibody**	**Sigma Aldrich, T5168**	**mouse, monoclonal**	**1:10000**
**β-Actin**	**Anti-β-actin antibody**	**Sigma Aldrich A-5316**	**mouse, monoclonal**	**1:10000**

Supplementary table 2. Primers and probes used for gene amplification.

Gene	Direction	Primer sequence 5' ⟶ 3'
**18S**	**FWD**<br>**REV**<br>**PB**	CGG CTA CCA CAT CCA AGG AA<br>GCT GGA ATT ACC GCG GCT<br>GAC GGC AAG TCT GGT GCC AGC A
**HPRT**	**FWD**<br>**REV**<br>**PB**	AGCCGACCGGTTCTGTCAT<br>GGTCATAACCTGGTTCATCATCAC<br>CGACCCTCAGTCCCAGCGTCGTGAT
**ACC**	**FWD**<br>**REV**<br>**PB**	TGGGCGGGATGGTCTCTTT<br>AGTCGCAGAAGCAGCCCATT<br>ACCTTTGAAGATTTCGTCAGGATCTTTGATGA
**AgRP**	**FWD**<br>**REV**<br>**PB**	GCA CAA GTG GCC AGG AAC TC<br>CAG GAC ACA GCT CAG CAA CAT<br>CAA GCA TCA ACA AGC AAA GGC CAT GC
**β1-ADR**	**FWD**<br>**REV**<br>**PB**	CCCCAAGTGCTGCGATT<br>AGGTACACGAAGGCCATGATG<br>TCGTCCGTCGTCTCCTTCTACGTGC
**B2-ADR**	**FWD**<br>**REV**<br>**PB**	GTGGATCGCTATGTTGCTATCACA<br>CACTCGGGCCTTATTCTTGGT<br>CGCCCTTCAAGTACCAGAGCCTGCT
**B3-ADR**	**FWD**<br>**REV**<br>**PB**	CACCGCTCAACAGGTTTGATG<br>CCCAGAAGTCCTGCAAAAACG<br>ACGTGAAGGGCCGTGAAGATCCAGC
**CEBPα**		Commercial probe Assay ID:Rn00560963_s1
**FAS**		Commercial probe Assay ID: Mm01253292_m1
**LPL**	**FWD**<br>**REV**<br>**PB**	GGGAAATGATGTGGCCAGATT<br>CCCTAAGAGGTGGACGTTGTCT<br>ACTGGATGGAAGGAGGAGGAGTTTAACTACCCCC
**PPARγ**	**FWD**<br>**REV**<br>**PB**	TGGAGTCCACGCATGTGAAG<br>CGCCAGCTTTAGCCGAATAG<br>CTGCAAGGGCTTCTTTCGGCGA
**SF-1**		Commercial primers provided by Applied Biosystems<br>(Ref. Rn00584298_m1)
**UCP3**		Commercial primers provided by Applied biosystems<br>(Ref. Rn00565874 m1)

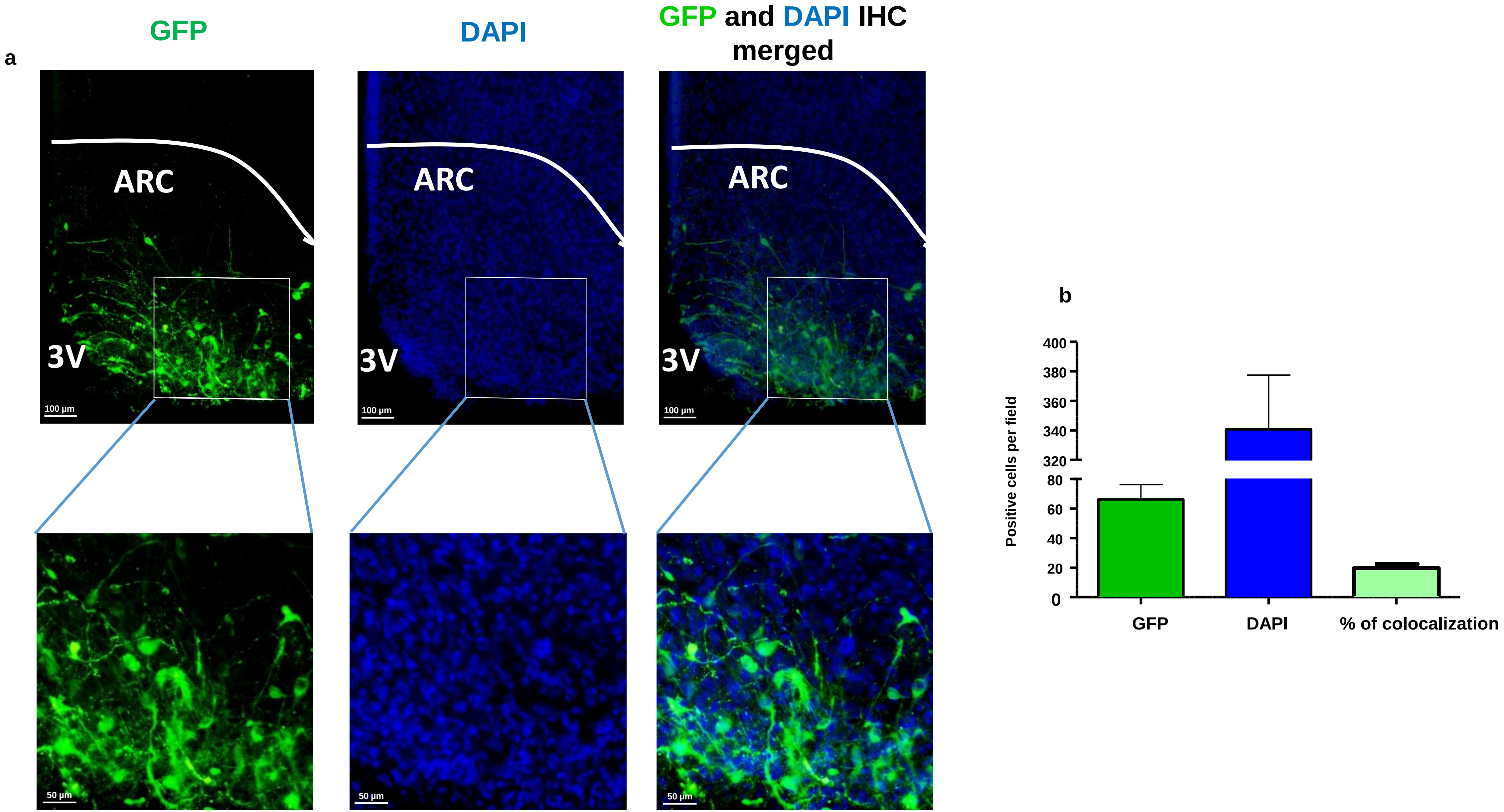

Supplementary Figure 1. Infection capacity and cells transducers after virogenetic inhibition of p53 into the ARC. Representative immunofluorescence showing GFP and DAPI expression in the ARC of rats after injection of Ad-DNp53RK in the ARC. Note the co-localization merged between GFP and DAPI (a). Number of GFP and DAPI positive cells and % of co-localization per field (b). Green and blue fluorescence staining represent GFP and DAPI reactivity, respectively. Dash lines indicate ARC boundaries. Abbreviations: third ventricle (3V), hypothalamic arcuate nucleus (ARC),immunohistochemistry (IHC). Values are mean ± SEM of 9-11 fields per group.

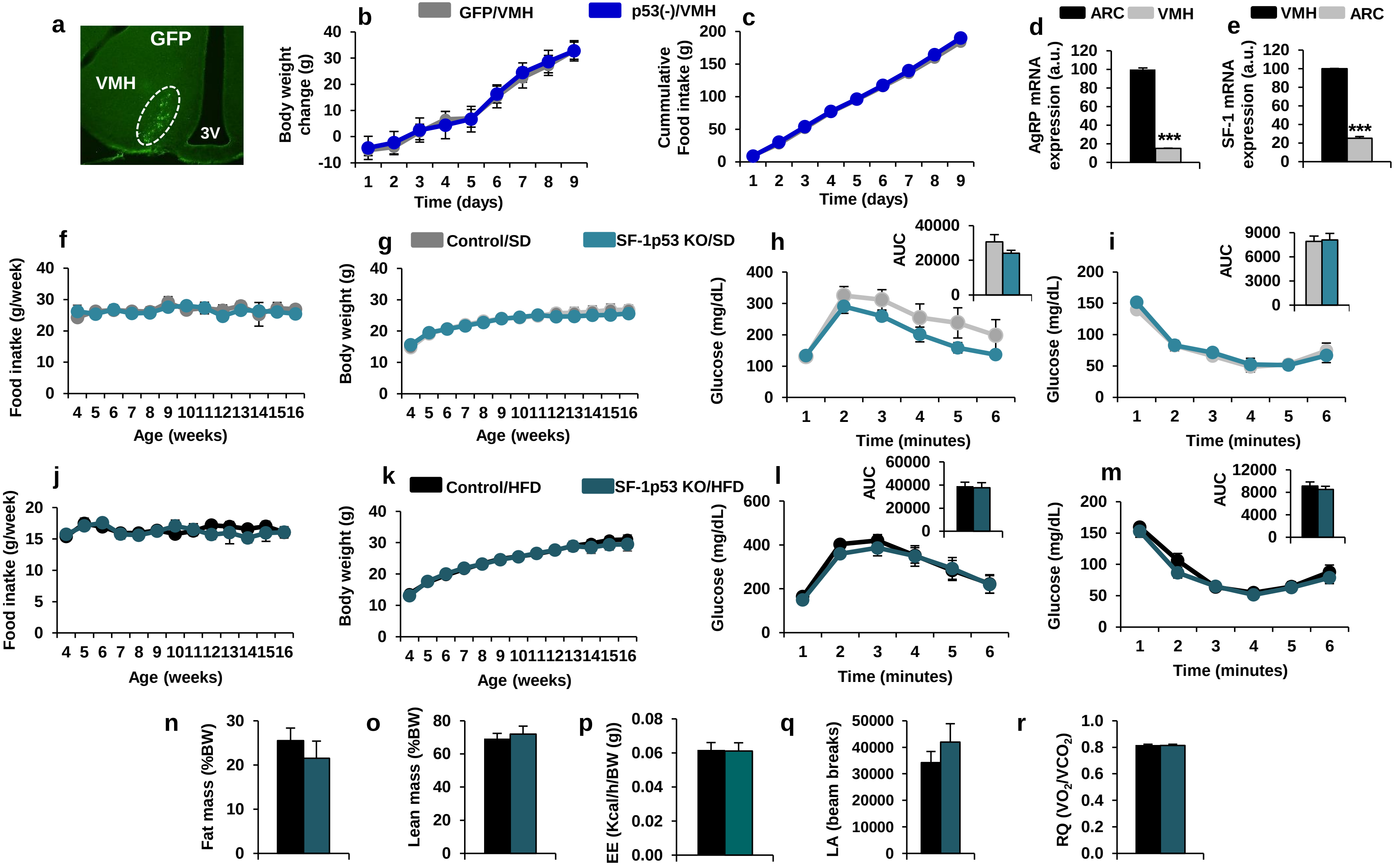

Supplementary Figure 2. Deletion of p53 in the VMH does not alter energy balance. Representative immunofluorescence showing GFP expression in the VMH (a). Body weight change (b) and cumulative food intake (c) of rats after 9 days of the injection of Ad-GFP and Ad-DNp53RK in the VMH. mRNA expression of AgRP (d) and SF1 (e) in the hypothalamic arcuate nucleus (ARC) and ventromedial nucleus (VMH). Food intake (f); body weight (g); glucose tolerance test (h); and insulin tolerance test (i) in control and SF1-Cre p53*loxP/loxP* mice fed a chow diet. Food intake (j); body weight change (k), glucose tolerance test (l); insulin tolerance test (m); fat mass (n); lean mass (o); energy expenditure (p); locomotor activity (LA) (q); and respiratory quotient (RQ) (r) in control and SF1-Cre p53$^{loxP/loxP}$ mice fed a HFD. Values are mean ± SEM of 7-11 animals per group. $^{*}P < 0.05$; $^{**}P < 0.01$; $^{***}P < 0.001$.

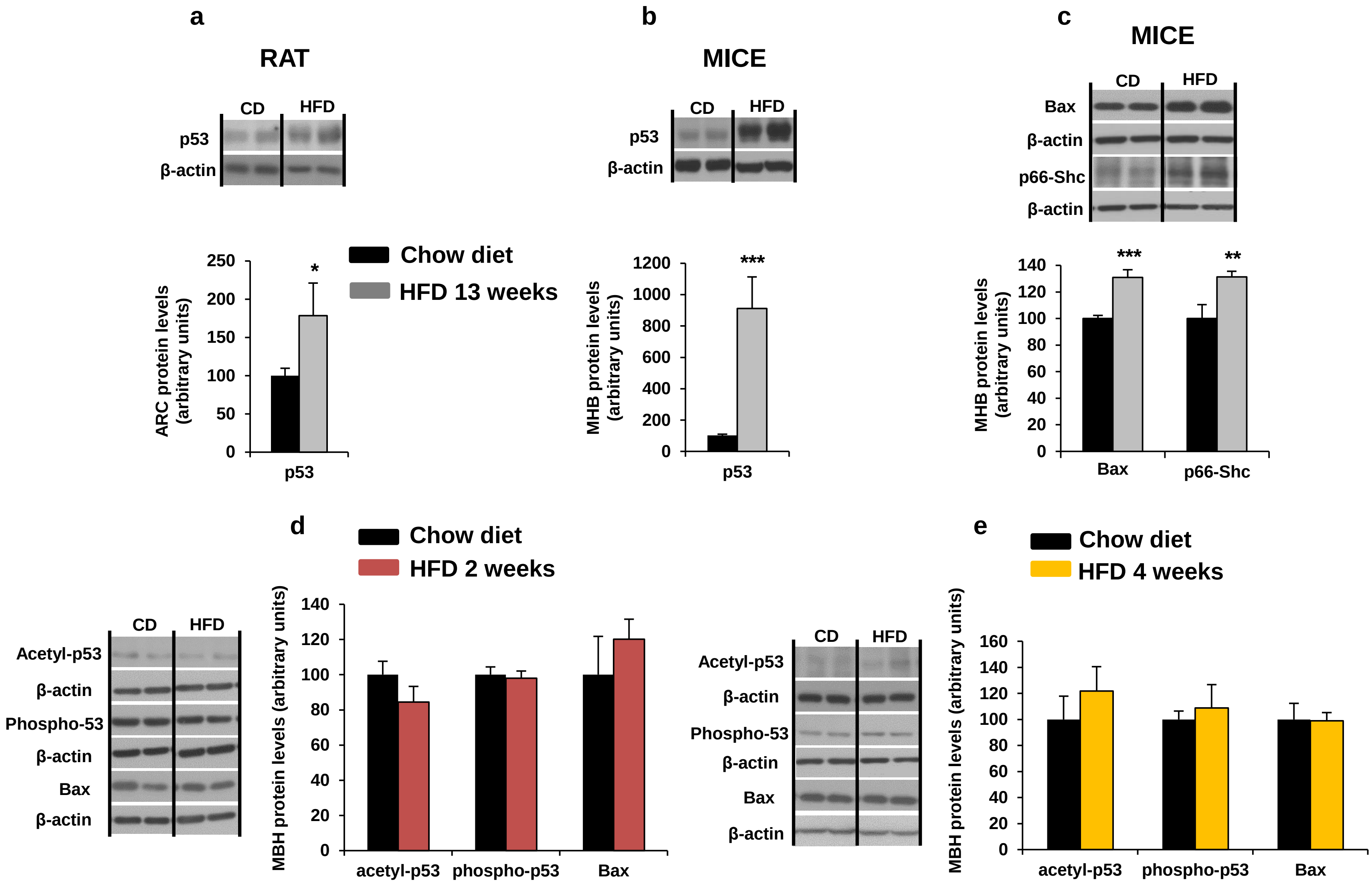

Supplementary figure 3. Effect of high fat diet in hypothalamic p53. Protein levels of p53 in the ARC of rats fed a chow diet (CD) or high fat diet (HFD) during 13 weeks (a). Protein levels of p53 in the mediobasal hypothalamus of mice fed a CD or HFD for 13 weeks (b). Protein levels of Bax and p66-Shc in the mediobasal hypothalamus of mice fed a CD or HFD for 13 weeks (c). Protein levels of acetyl-p53, phosphorylated levels of p53 and Bax in the mediobasal hypothalamus of mice fed a CD or HFD for 2 weeks (d) and for 4 weeks (e). β-actin was used to normalize protein levels. Dividing lines indicate spliced bands from the same gel. Values are mean ± SEM of 5-7 animals per group. *$P < 0.05$; **$P < 0.01$; ; ***$P < 0.001$.

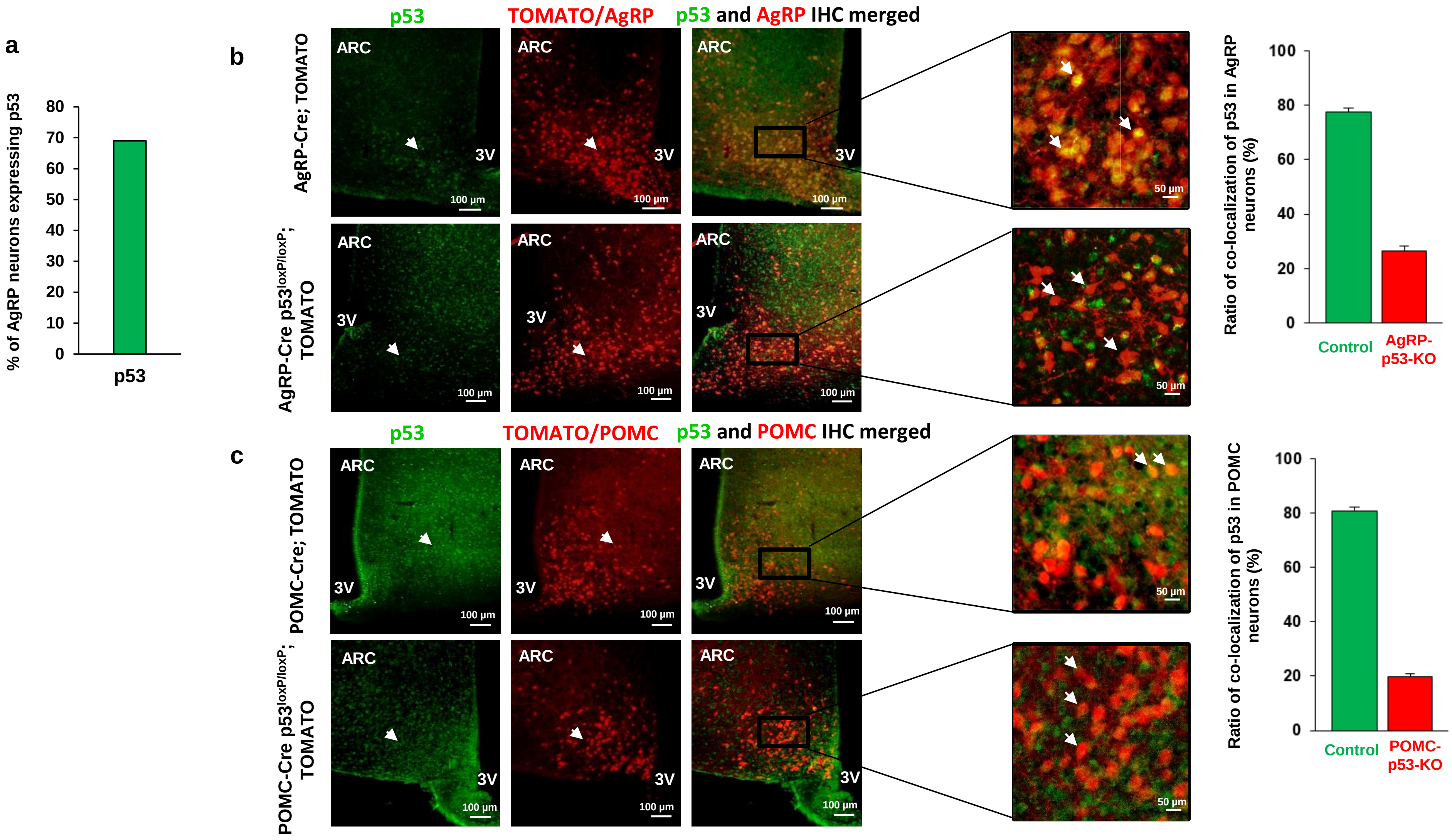

Supplementary figure 4. Deletion of p53 is restricted to AgRP and POMC neurons. FACS sorting and single-cell RNA sequencing of AgRP-eGFP neurons showing p53 expression (GEO Database repository: GEO Accession: GSE92707) (a). Representative photomicrographs of brain slices from *AgRP*-Cre; Tomato (control) or AgRP-Cre p53loxP/lox; Tomato (AgRPp53 KO) (b) and *POMC*-Cre; Tomato (control) or POMC-Cre p53loxP/lox; Tomato (POMCp53 KO) (c). Green flourescence staining and red fluorescence represent p53 and AgRP or POMC immunoreactivity, respectively. Arrows indicate AgRP and POMC neurons. Note the co-localization between p53 and AgRP in AgRP-Cre; Tomato control brain (b). This co-localization is markedly reduced in AgRP neurons of AgRP-Cre p53loxP/loxP mice. Note the co-localization between p53 and POMC in POMC-Cre; Tomato control brain (c). This co-localization is markedly reduced in POMC neurons of POMC-Cre p53loxP/loxP mice. Tomato brain (c). Dash lines indicate ARC boundaries. Abbreviations: third ventricle (3V), hypothalamic arcuate nucleus (ARC), immunohistochemistry (IHC).

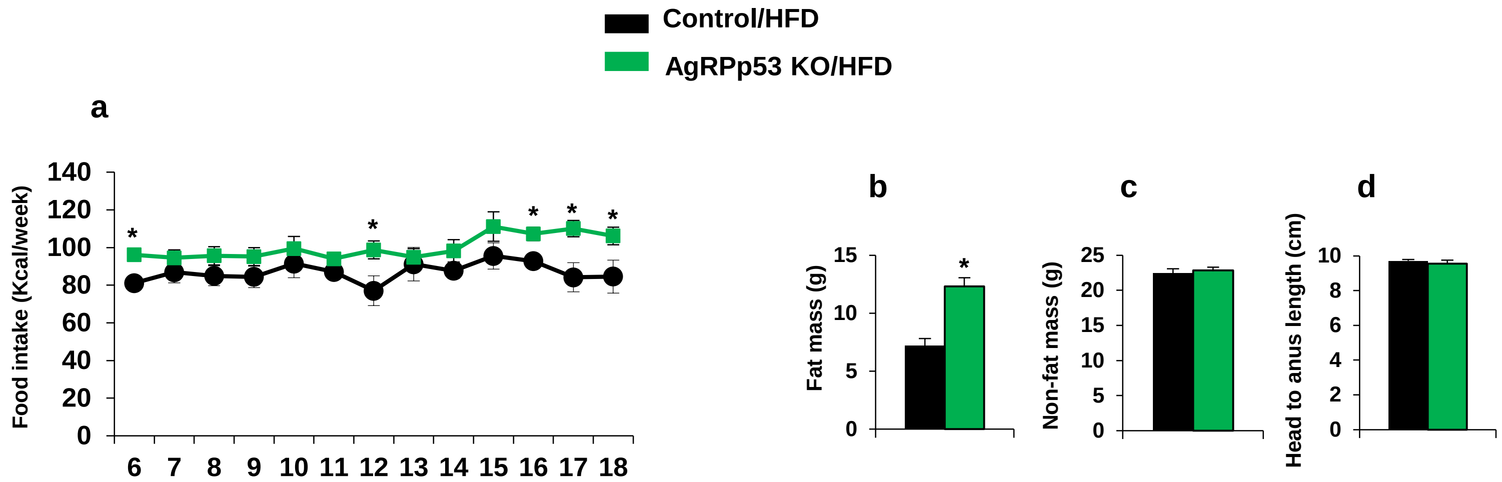

Supplementary Figure 5. Food intake, adiposity and body length. Weakly energy intake (a); fat mass (g) (b); non-fat mass (g) (c) and body length from head to anus (d) of control and AgRPp53 KO mice fed a HFD for 13 weeks. Values are mean ± SEM of 5-10 animals per group. *P < 0.05.

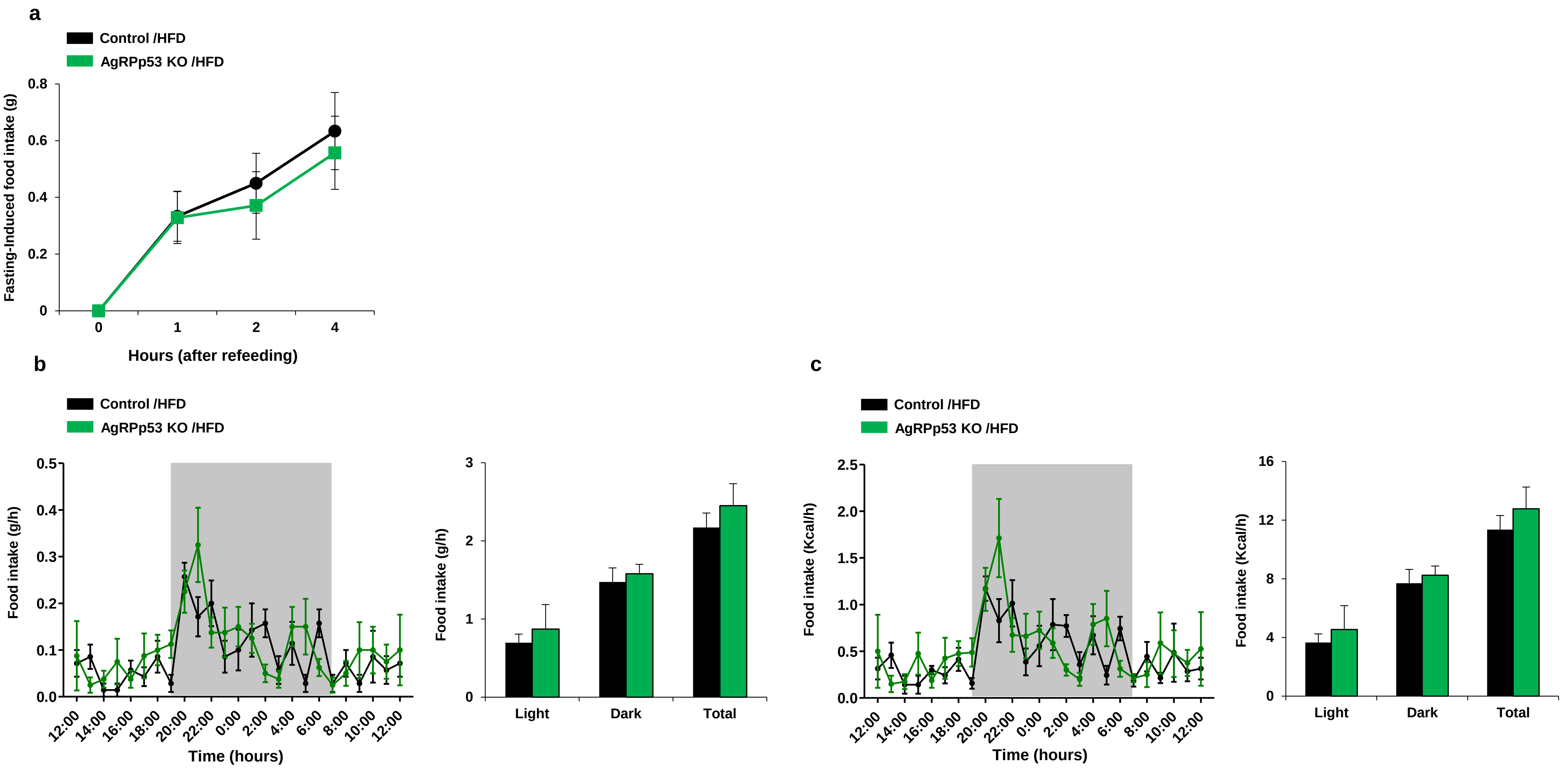

Supplementary Figure 6. Fasting-induced refeeding and circadian cycles. Food intake of control and AgRPp53 KO mice fed a HFD fasted overnight and then refed for 4 hours (a). Feeding pattern of control and AgRPp53 KO mice fed a HFD for 10 weeks over the 24-hour cycle during light and dark periods. Food intake represented as (g/h) (b) or (Kcal/h) (c). Values are mean ± SEM of 5-8 animals per group.

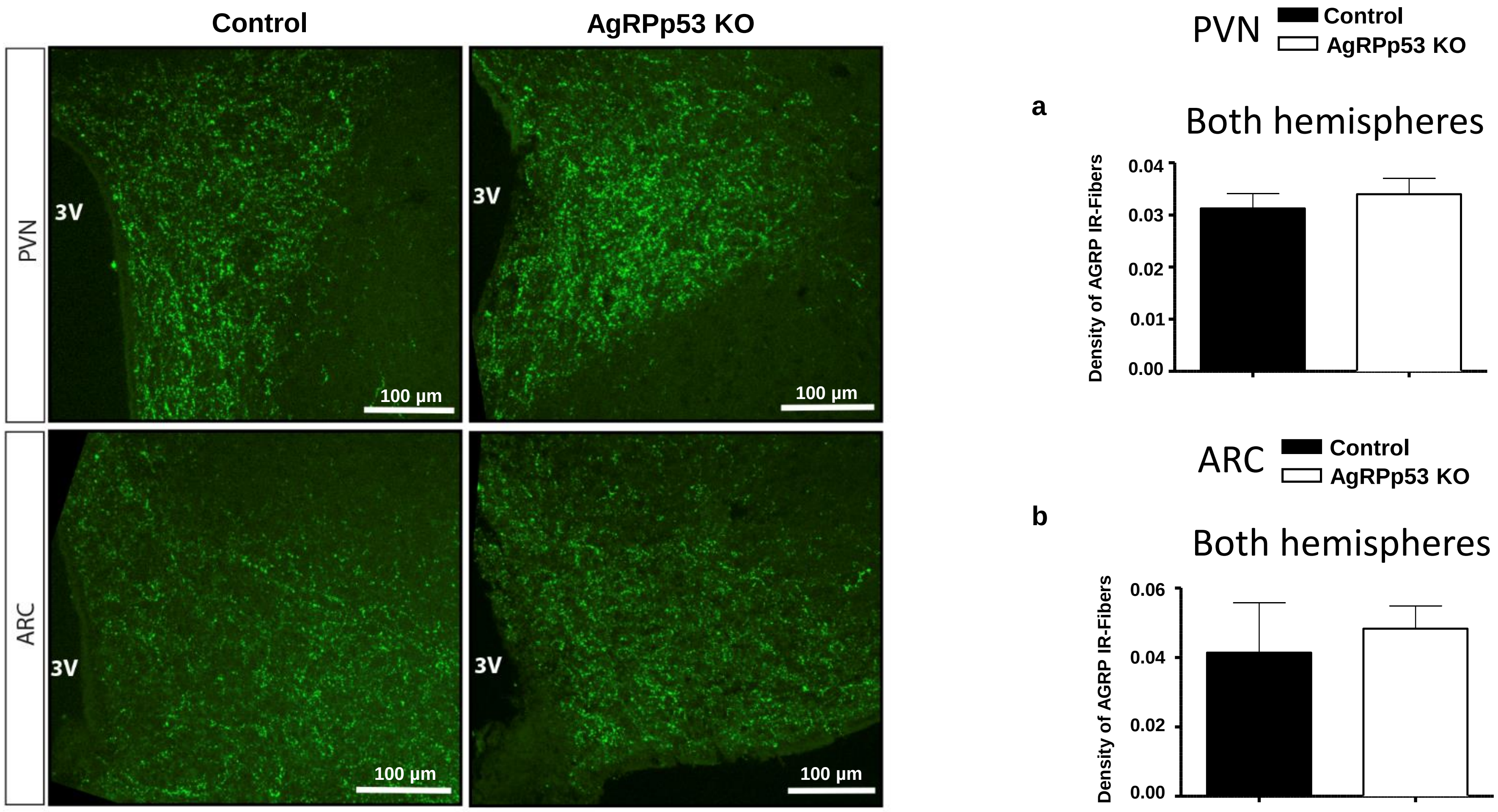

Supplementary Figure 7. AgRP fiber density is similar between Control and AgRPp53 KO mice. Representative immunofluorescence images showing AgRP staining in the PVN (a) and ARC (b) of control and AgRPp53 KO mice fed a HFD for 13 weeks and integrated density quantification. Values are mean ± SEM of 6-26 fields per group. Abbreviations: third ventricle (3V), hypothalamic arcuate nucleus (ARC), hypothalamic paraventricular nucleus (PVN).

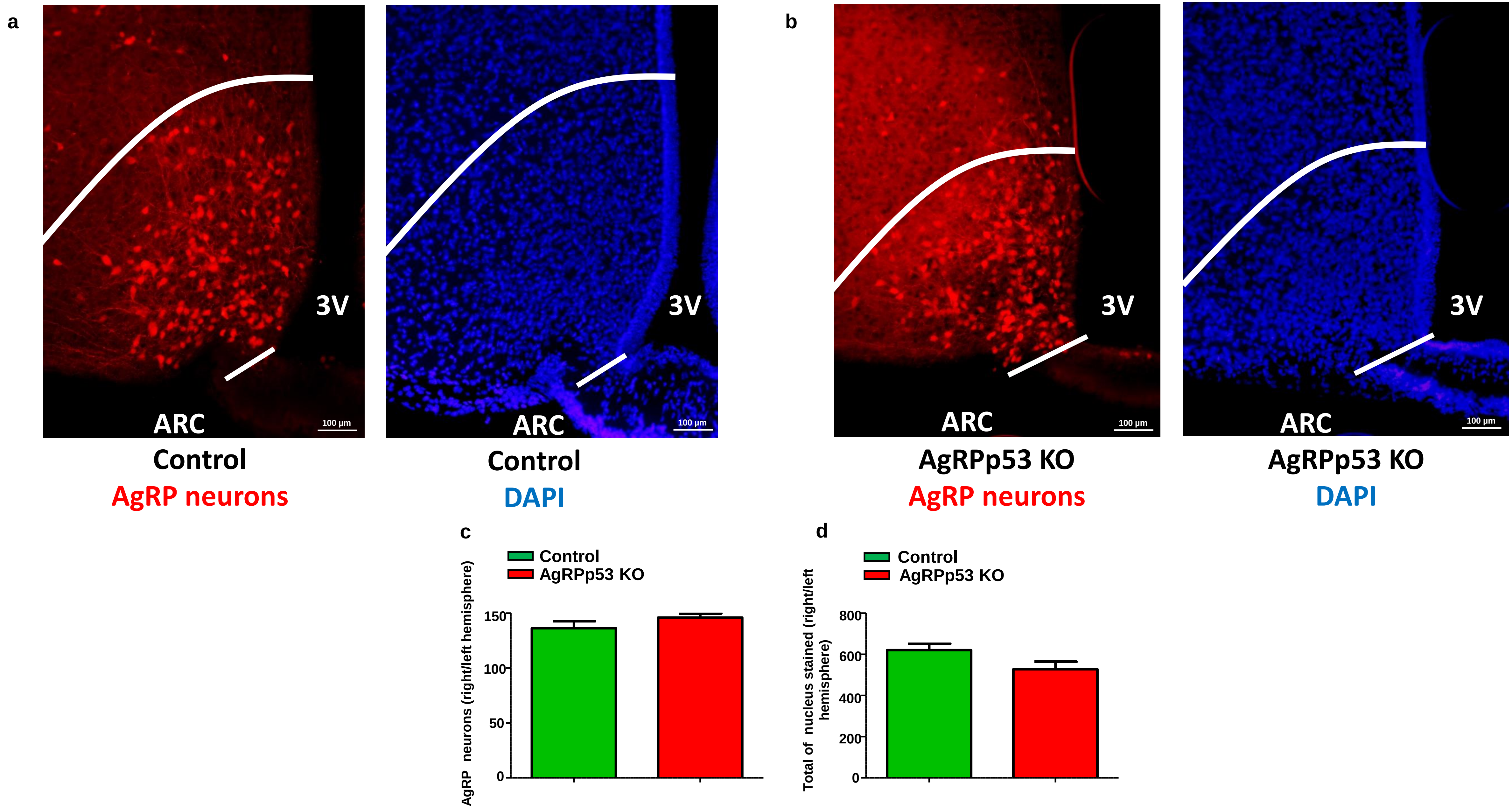

Supplementary Figure 8. Number of AgRP neurons in control and AgRPp53 KO mice. Representative immunofluorescence showing AgRP neurons and DAPI in the ARC of control (a) and AgRPp53 KO mice (b). Number of AgRP and DAPI positive cells in the ARC of control (c) and AgRPp53 KO mice (d). Red and blue fluorescence staining represents AgRP and DAPI reactivity, respectively. Dash lines indicate ARC boundaries. Abbreviations: third ventricle (3V) and hypothalamic arcuate nucleus (ARC). Values are mean ± SEM of 7-16 fields per group.

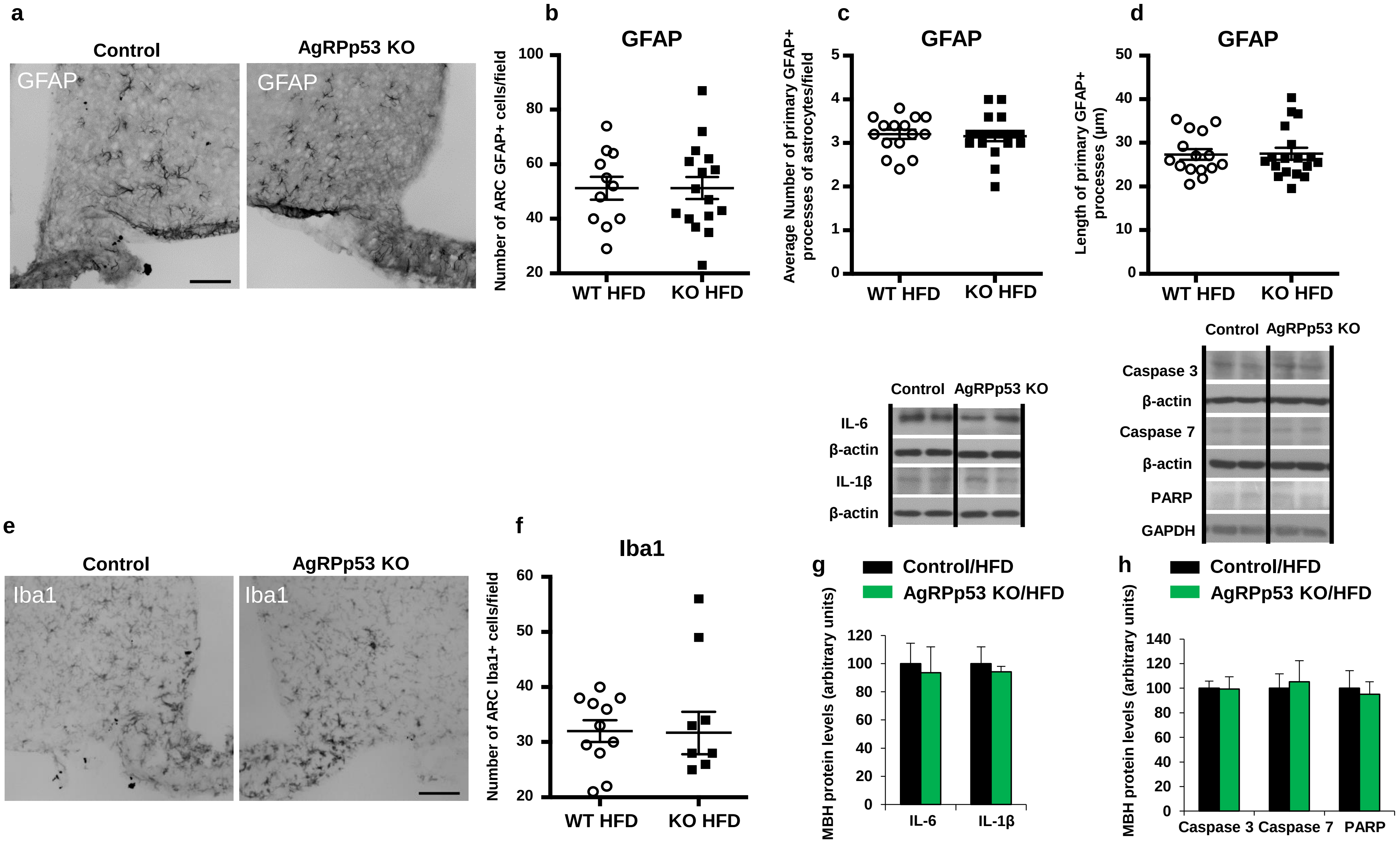

Supplementary Figure 9. Inflammation, apoptosis and senescence in AgRPp53 KO mice. Illustration of GFAP-ir astrocytes in the ARC (a); quantification of the number of GFAP-ir astrocytes (b); number of primary GFAP+ processes of astrocytes; (c) length of primary GFAP+ processed (d) per field in the ARC from control and AgRPp53 KO mice fed a HFD for 13 weeks. Illustration of Iba1-ir microglia in the ARC in the ARC (e); quantification of the number of iba1-ir microglia per field in the ARC (f) from control and AgRPp53 KO mice fed a HFD for 13 weeks. MBH protein levels of IL-6 and IL-1 β (g) and caspase 3, caspase 7 and PARP (h) from control and AgRPp53 KO mice fed a HFD for 13 weeks. β-actin and GAPDH were used to normalize protein levels. Dividing lines indicate spliced bands from the same gel. nValues are mean ± SEM of 8-11 mice per group.

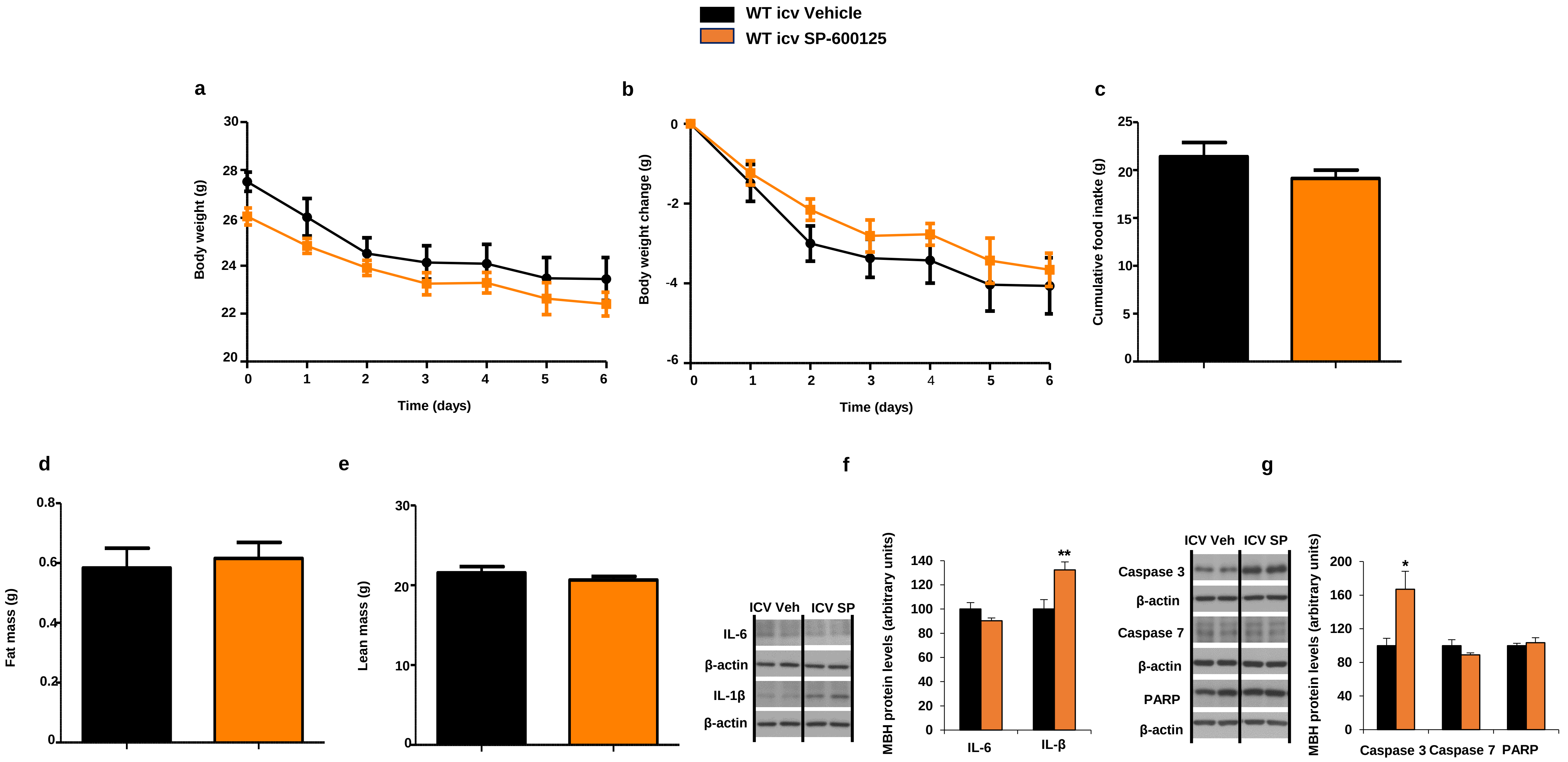

Supplementary Figure 10. The JNK inhibitor SP-600125 did not modify body weight, food intake or adiposity in control mice fed a chow diet. Body weight (a); body weight change (b); cumulative food intake (c); fat mass (d); non-fat mass (e) MBH protein levels of IL-6 and IL-1 β (f); MBH protein levels of caspase 3, caspase 7 and PARP (g); of mice after i.c.v. injection of saline or SP-600125 (1.5 µg/mouse) during 6 days. β-actin was used to normalize protein levels. Dividing lines indicate spliced bands from the same gel. Values are mean ± SEM of 6-10 animals per group. *P < 0.05; **P < 0.01.

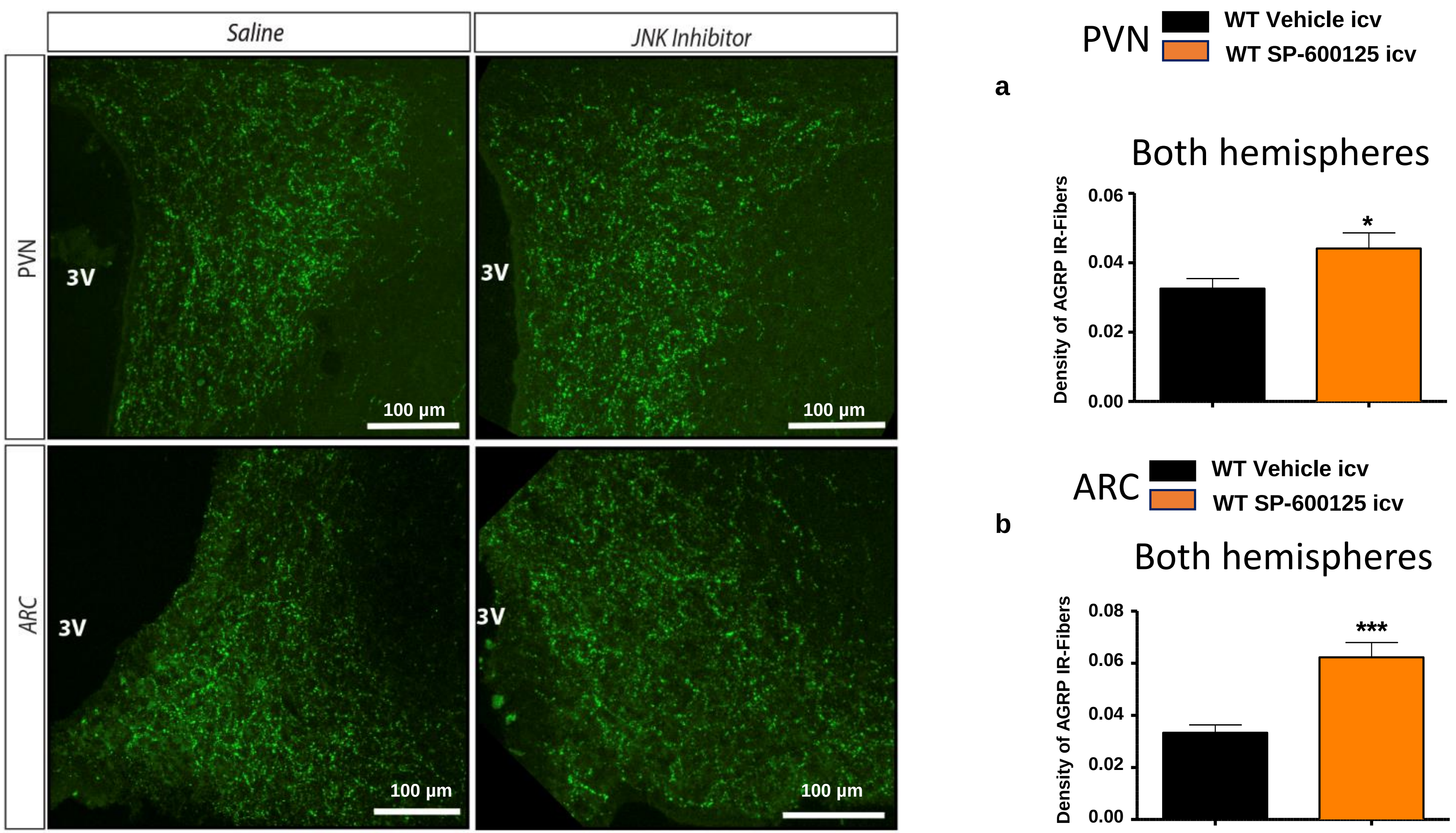

Supplementary Figure 11. The JNK inhibitor SP-600125 did not affect AgRP fiber density in the ARC and PVN. Representative immunofluorescence images showing AgRP staining in the PVN (a) and ARC (b) of control mice after i.c.v. injection of saline or JNK inhibitor SP-600125 and integrated density quantification (a-b). Values are mean ± SEM of 8-24 fields per group. *P < 0.05; **P < 0.01; ***P< 0.001. Abbreviations: third ventricle (3V), hypothalamic arcuate nucleus (ARC), hypothalamic paraventricular nucleus (PVN).

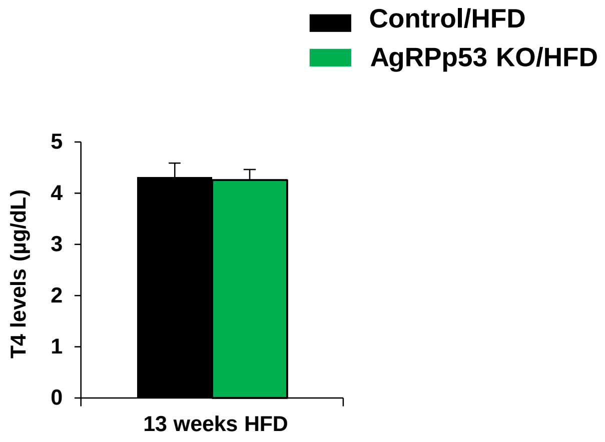

Supplementary Figure 12. Circulating T4 levels were similar between control and AgRPp53 KO mice. Levels of thyroxine (T4) in control and AgRPp53 KO mice fed a HFD for 13 weeks. Values are mean ± SEM of 6-10 animals per group.

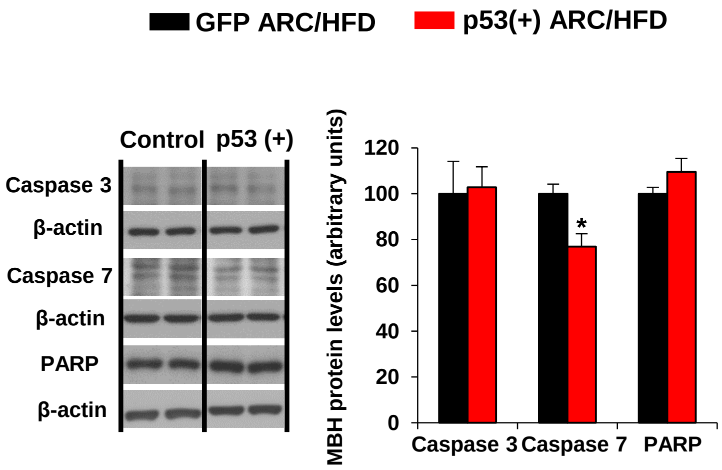

Supplementary Figure 13. Apoptosis and senescence markers in DIO mice after over-expression of p53 in ARC. MBH protein levels of caspase 3, caspase 7 and PARP from DIO mice after injection of Ad-GFP or Ad-p53 in the ARC during 7 days. β-actin was used to normalize protein levels. Dividing lines indicate spliced bands fromnthe same gel. Values are mean ± SEM of 5-7 animals per group. *$P < 0.05$.

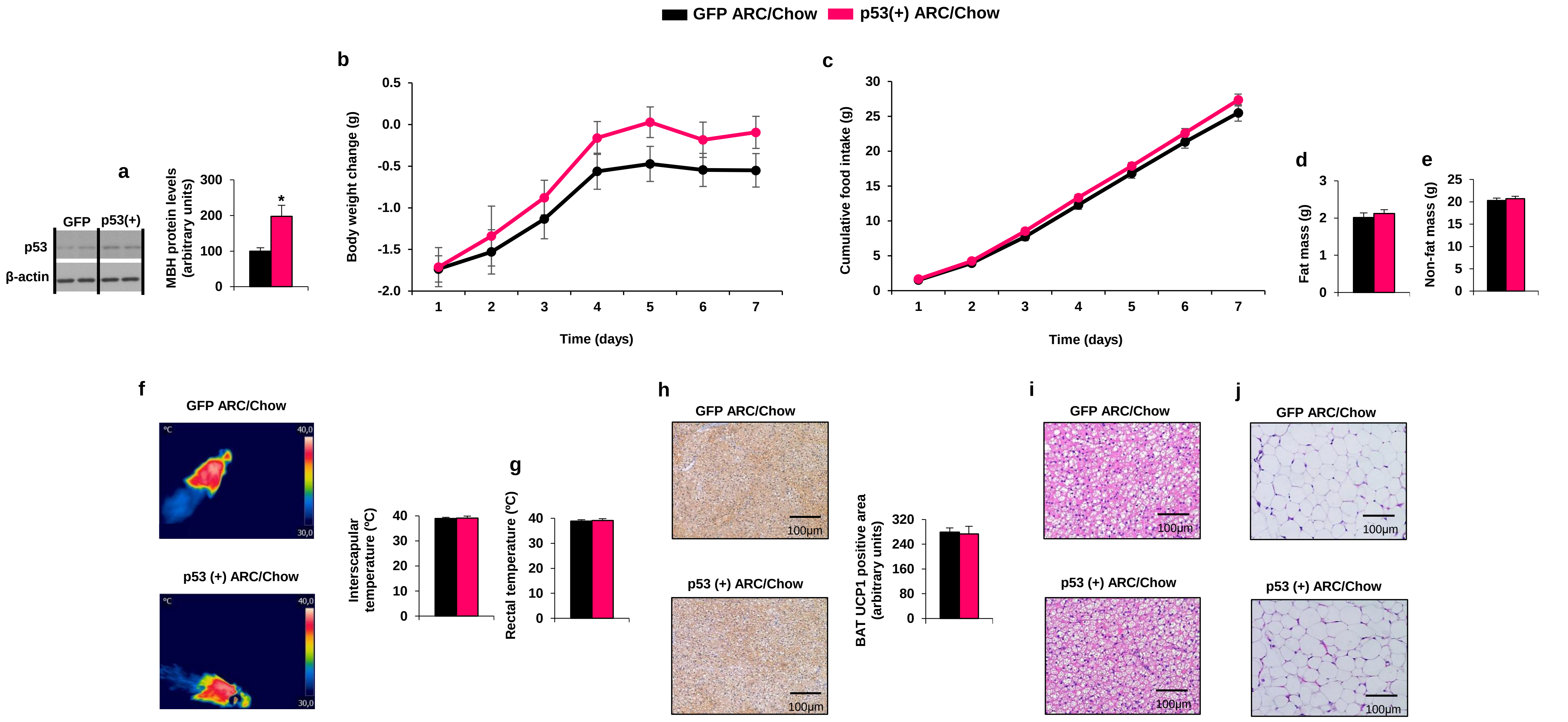

Supplementary Figure 14. The effects of hypothalamic p53 on energy balance are diet dependent. ARC protein levels of p53 (a); body weight change (b); cumulative food intake (c); fat mass (d); non-fat mass (e); representative infrared thermal images and temperature of the BAT (f); rectal temperature (g); BAT UCP1 immunostaining (h); representative pictures of BAT histology (hematoxylin-eosin) (i); representative pictures of WAT histology (hematoxylin-eosin) (j) from lean mice after injection of Ad-GFP or Ad-p53 in the ARC during 7 days. β-actin was used to normalize protein levels. Dividing lines indicate spliced bands from the same gel. Values are mean ± SEM of 7-18 animals per group. *P < 0.05.

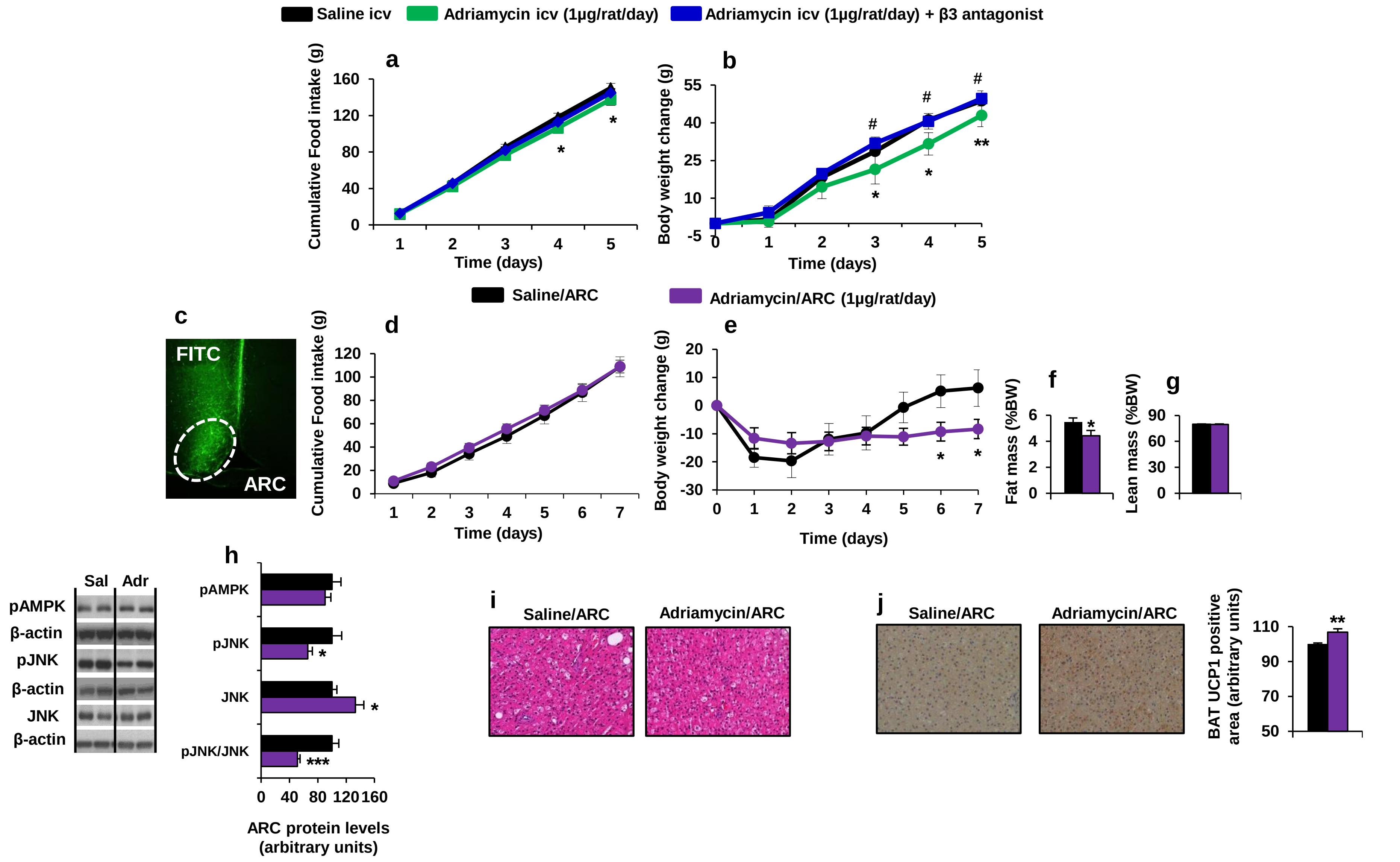

Supplementary figure 15. Effects of pharmacological stimulation of p53 with adriamycin on energy balance are mediated by the sympathetic nervous system. Effects of 5 days i.c.v. injections of vehicle, adriamycin (1 µg/rat/day) or SR59230A (3 mg/kg/day) in rats fed a high fat diet. Cumulative food intake (a) and body weight change (b). Fluorescein-isothio-cyanate (FITC) staining in the hypothalamic arcuate nucleus (ARC) (c); cumulative food intake (d); body weight (e); fat mass (f), lean mass (g); protein levels of pAMPK, pJNK and JNK in the ARC (h); representative BAT histology pictures (hematoxylin-eosin) (i), and BAT UCP1 immunostaining (j) of rats after the infusion of adriamycin in the ARC for 7 days. β-actin was used to normalize protein levels. Dividing lines indicate spliced bands from the same gel.Values are mean ± SEM of 7-15 animals per group. *$P < 0.05$; **$P < 0.01$.

Uncropped blots for Figures 1b and 1h

Related to Figure 1b

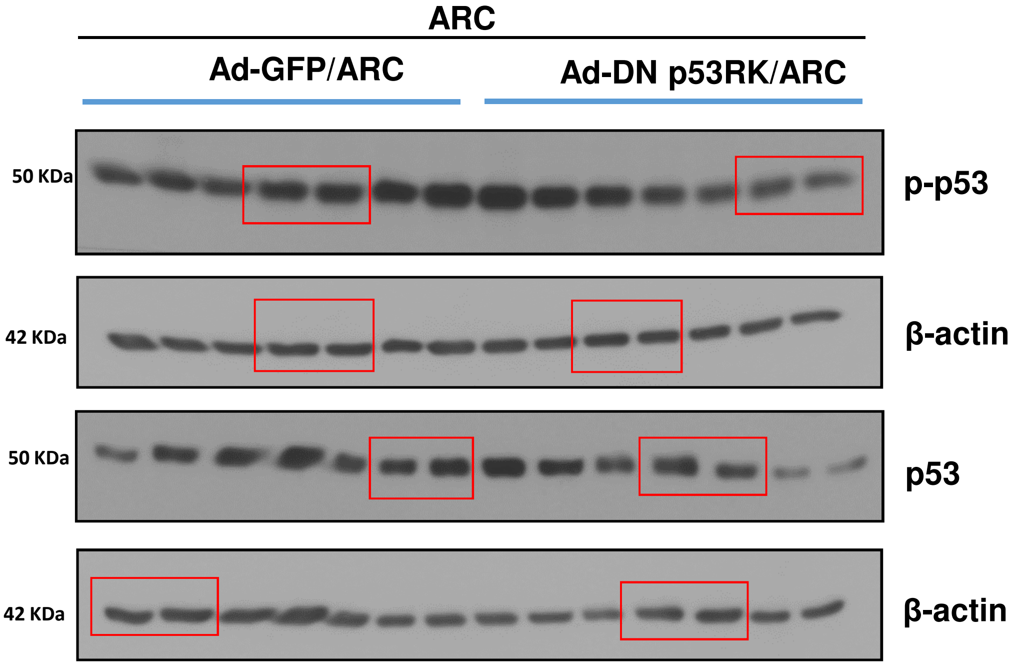

Related to Figure 1h

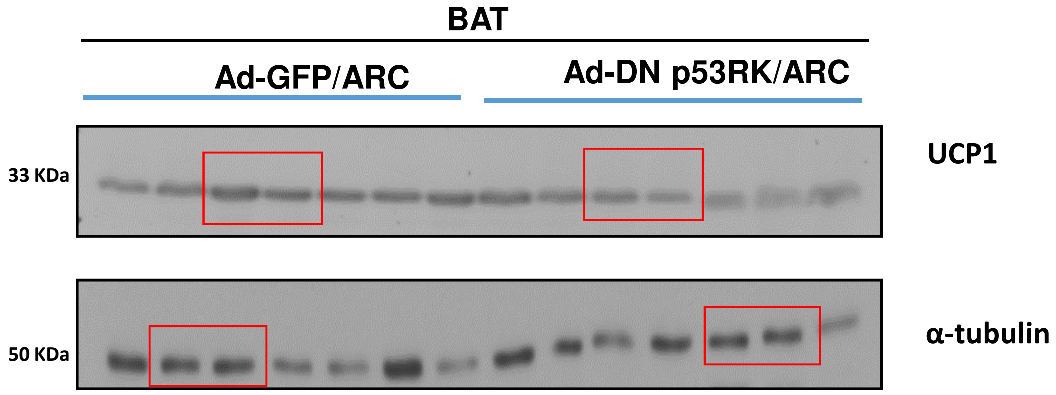

Supplementary figure 16. Appendix figure 1.

Uncropped blots for Figure 3k

Related to Figure 3k

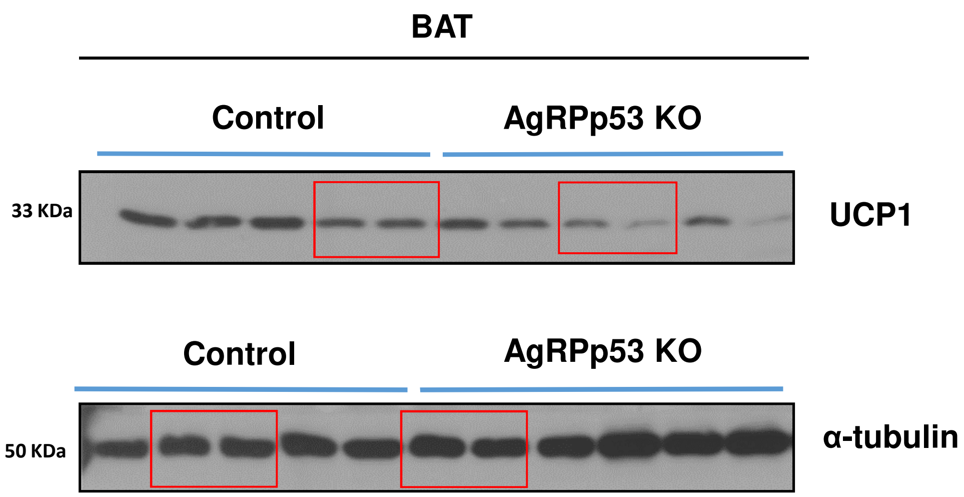

Supplementary figure 16. Appendix figure 3.

Uncropped blots for Figure 5a

Related to Figure 5a

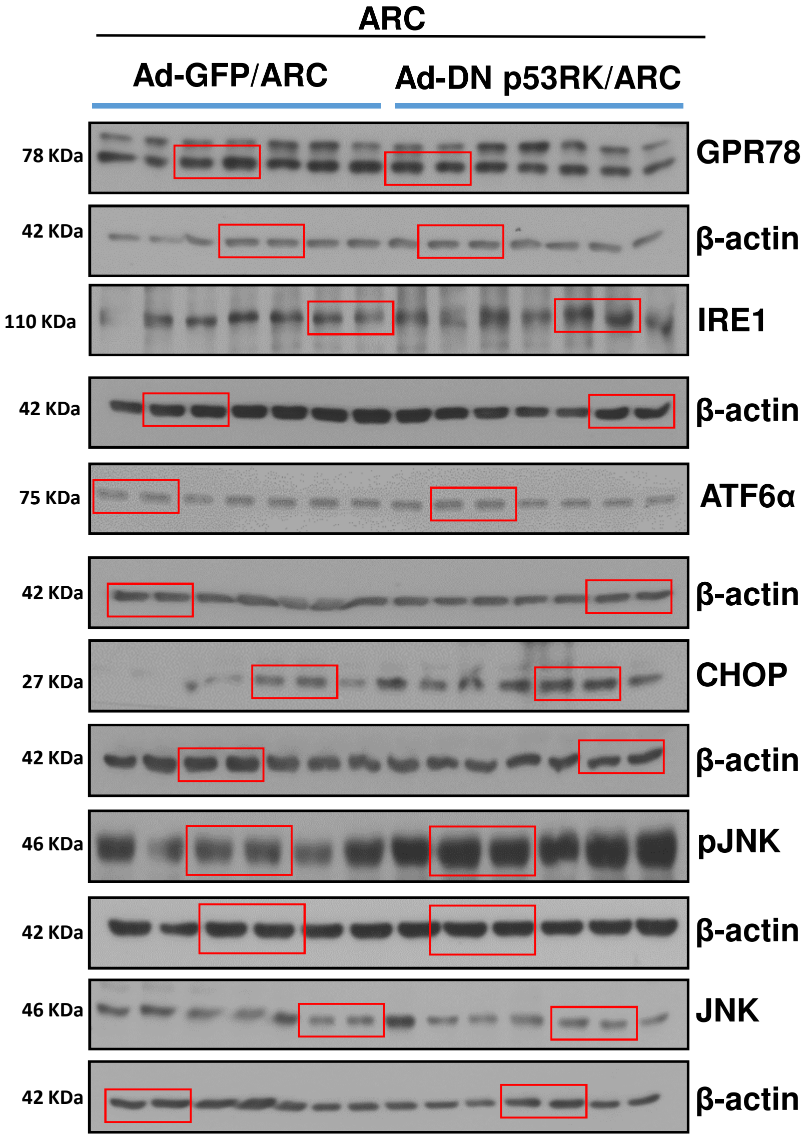

Supplementary figure 16. Appendix figure 5

Uncropped blots for Figure 5b

Related to Figure 5b

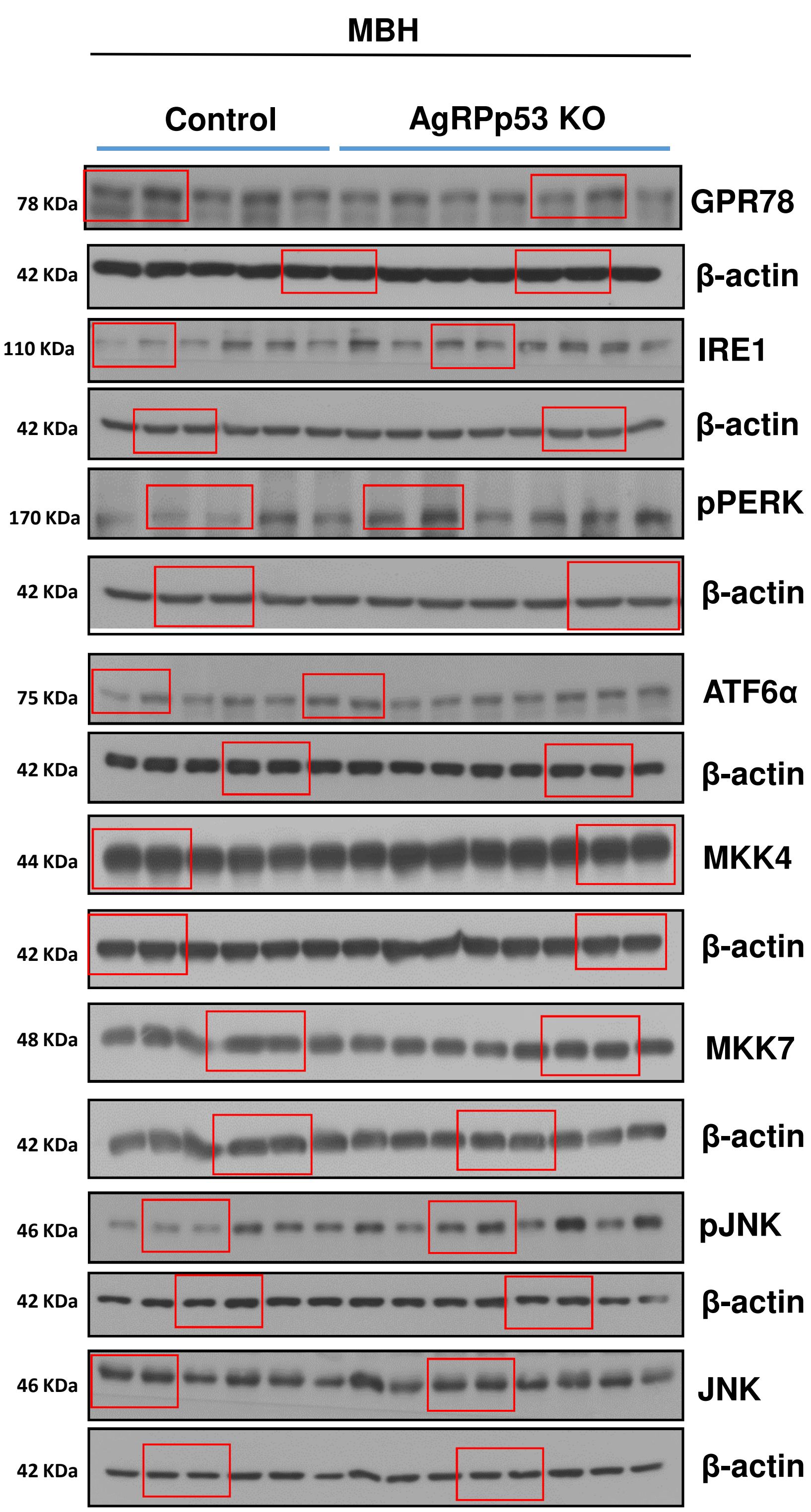

Supplementary figure 16. Appendix figure 5

Uncropped blots for Figure 6h and 6j

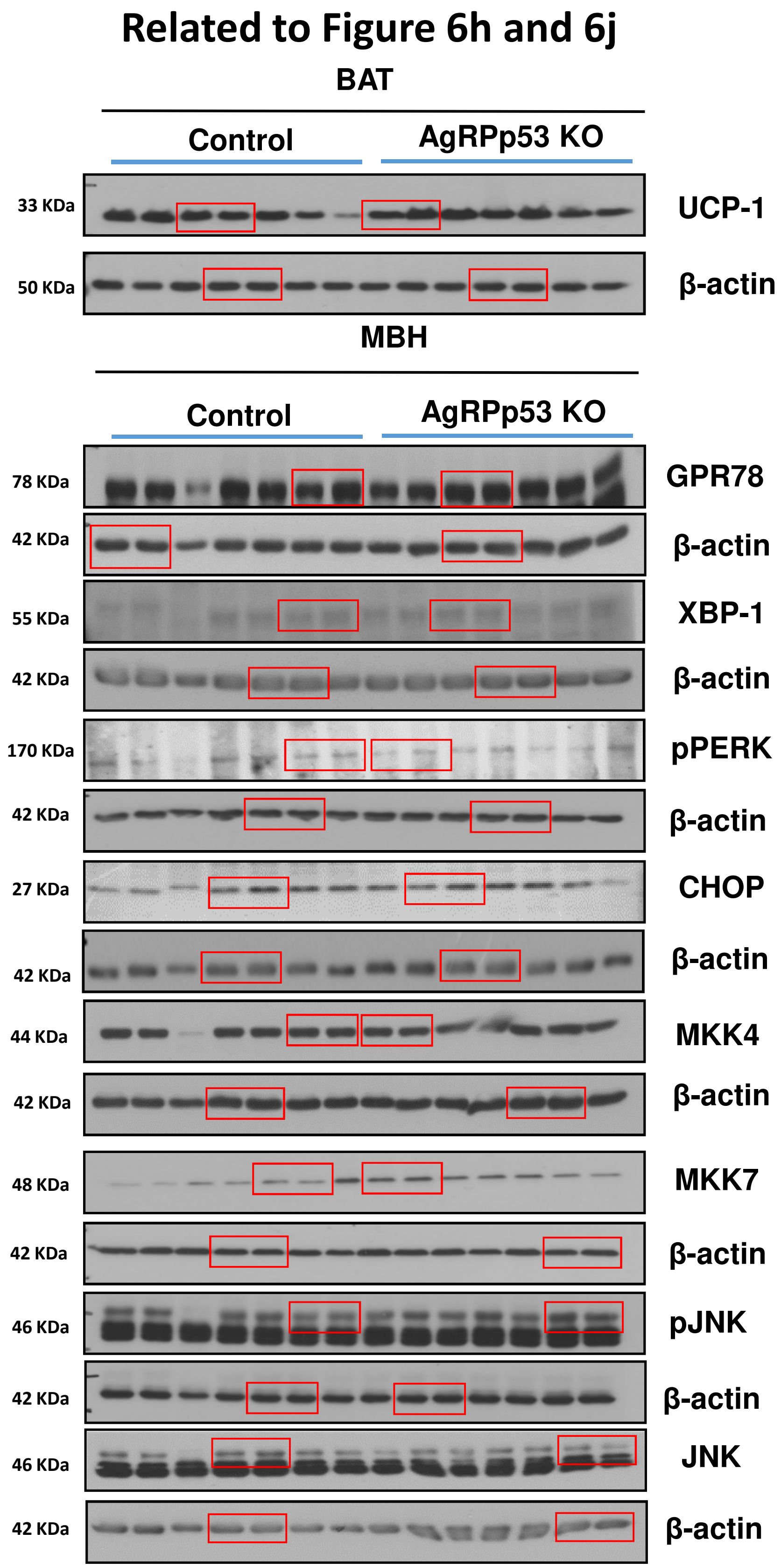

Supplementary figure 16. Appendix figure 6

Uncropped blots for Figure 7f, 7g, 7p and 7q

Related to Figure 7f and 7g

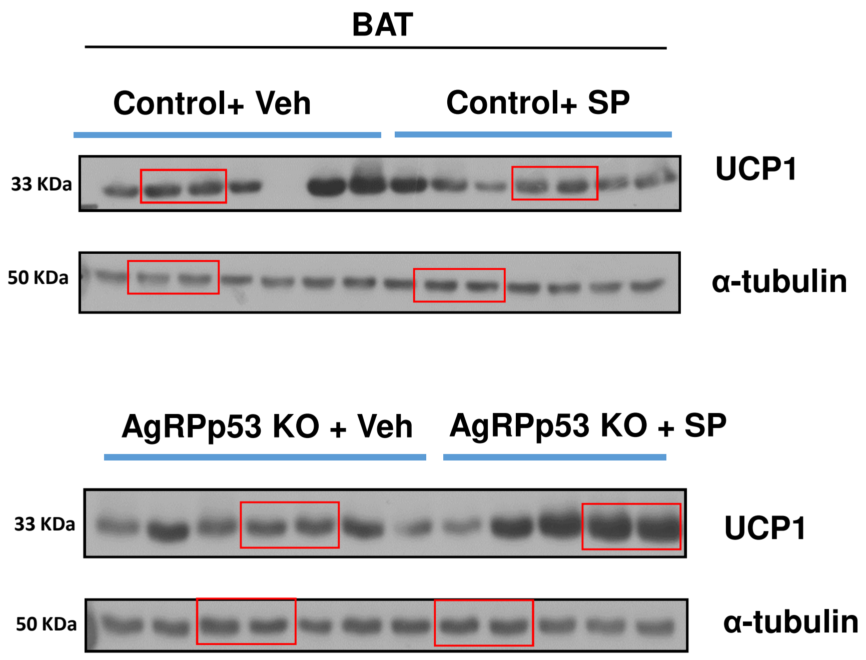

Related to Figure 7p and 7q

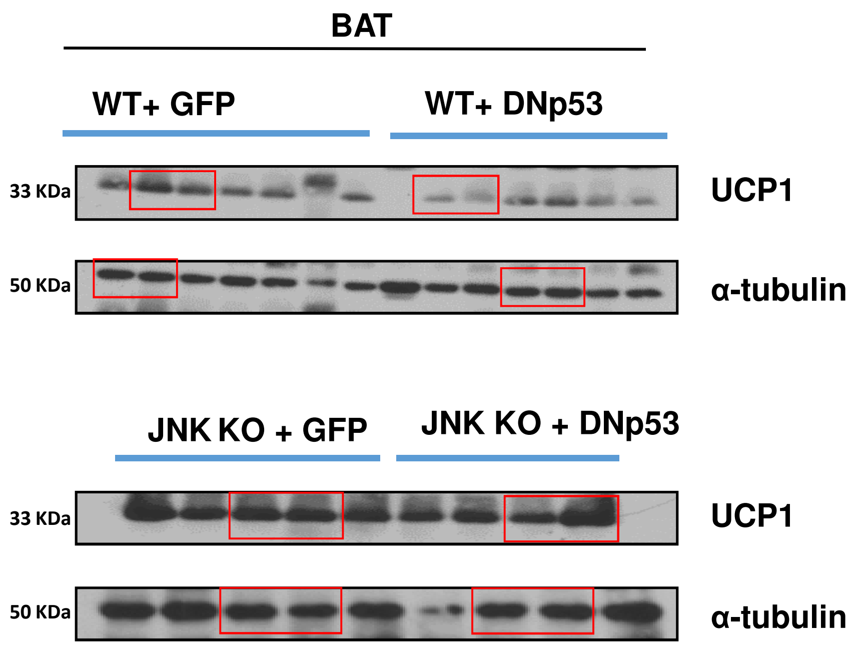

Supplementary figure 16. Appendix figure 7.

Uncropped blots for Figure 8b and 8o

Related to Figure 8b

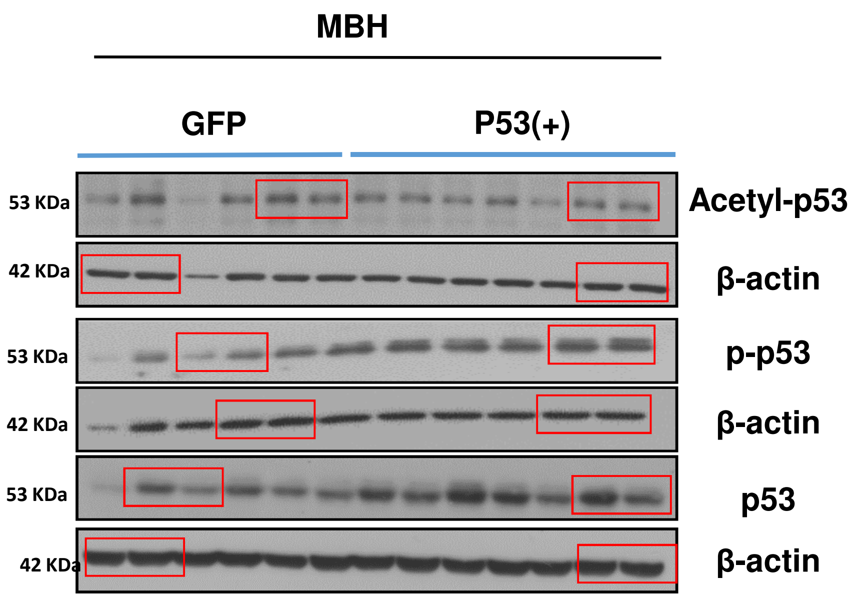

Related to Figure 8o

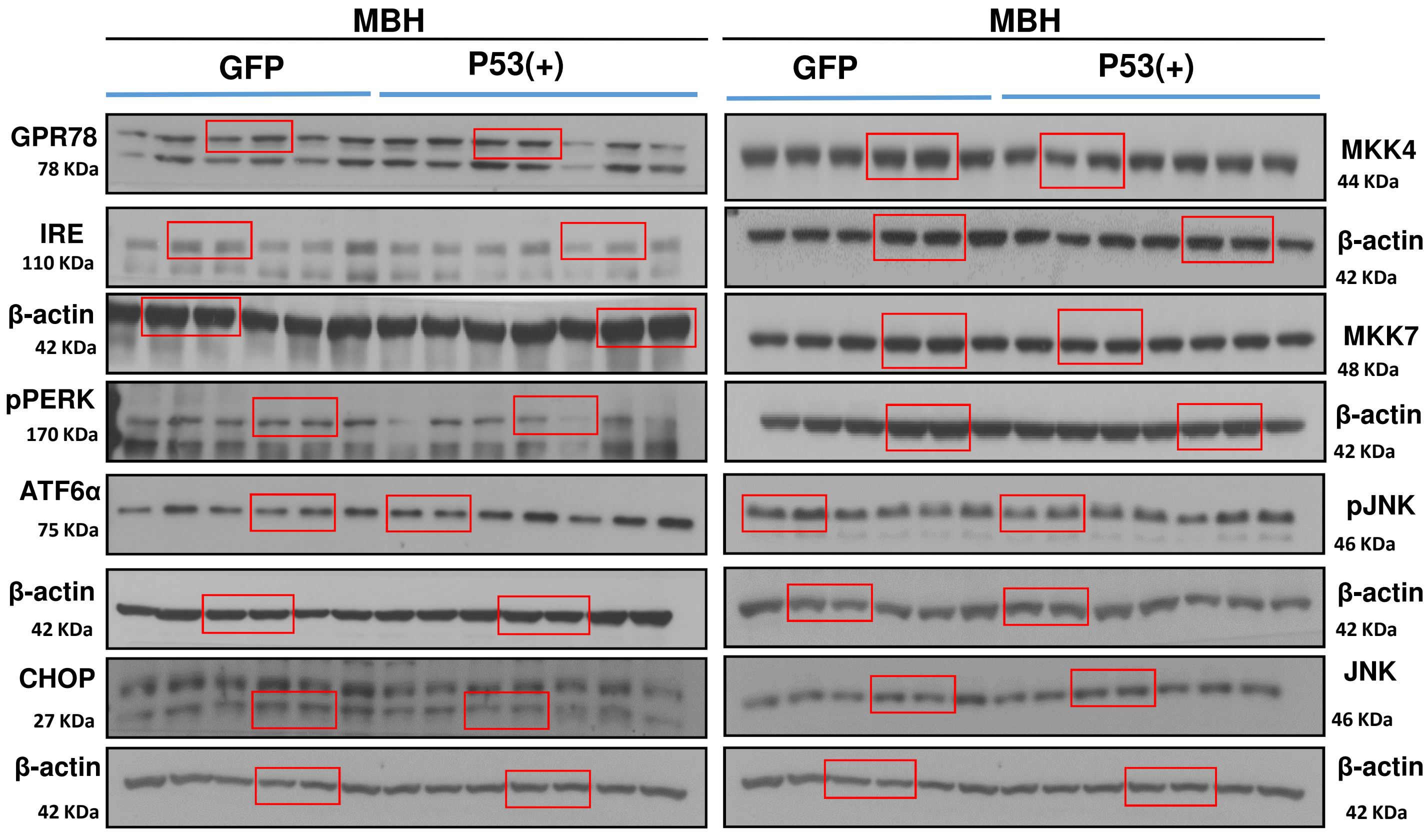

Supplementary figure 16. Appendix figure 8.

Uncropped blots for Figure S3a,S3b,S3c,S3d and S3e

Related to Figure S3a, S3b, S3c,S3d and S3e

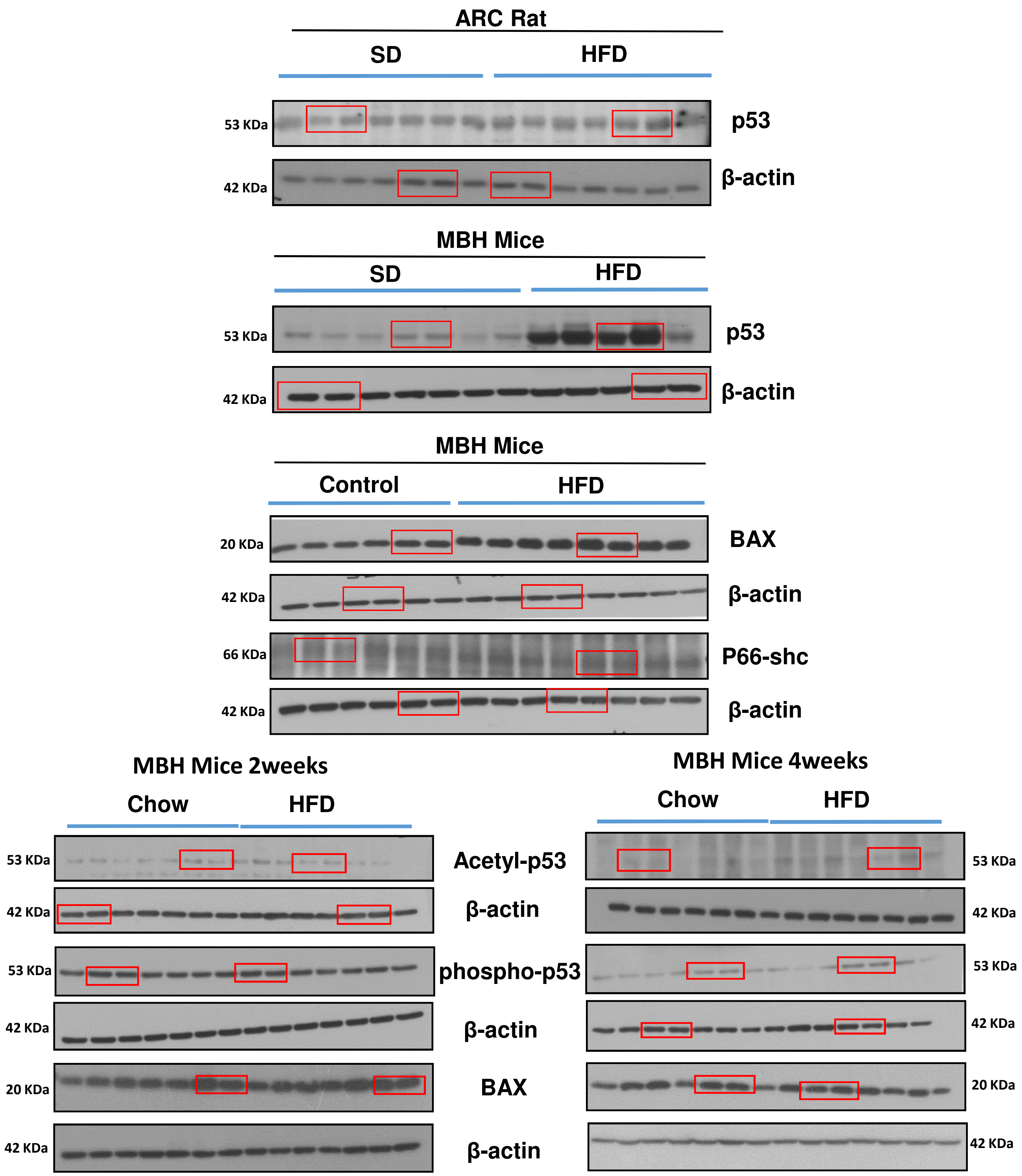

Supplementary figure 16. Appendix figure S3.

Uncropped blots for Figure S9g and S9h

Related to Figure S9g

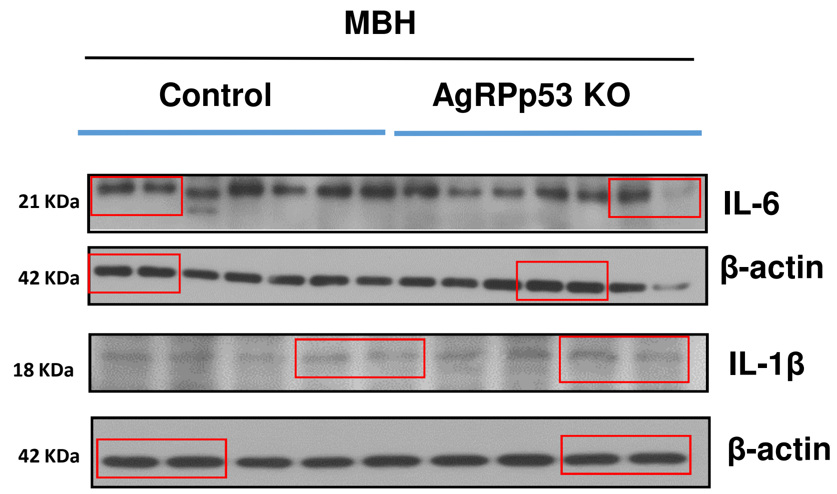

Related to Figure S9h

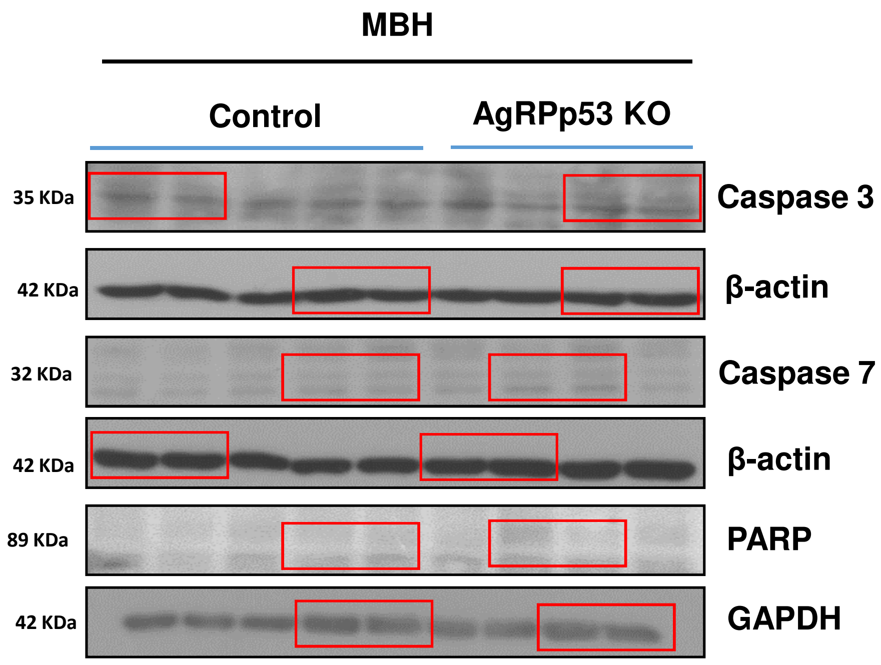

Supplementary figure 16. Appendix figure S9.

Uncropped blots for Suppl. Figure S10f and S10g

Related to Suppl. Figure S10f

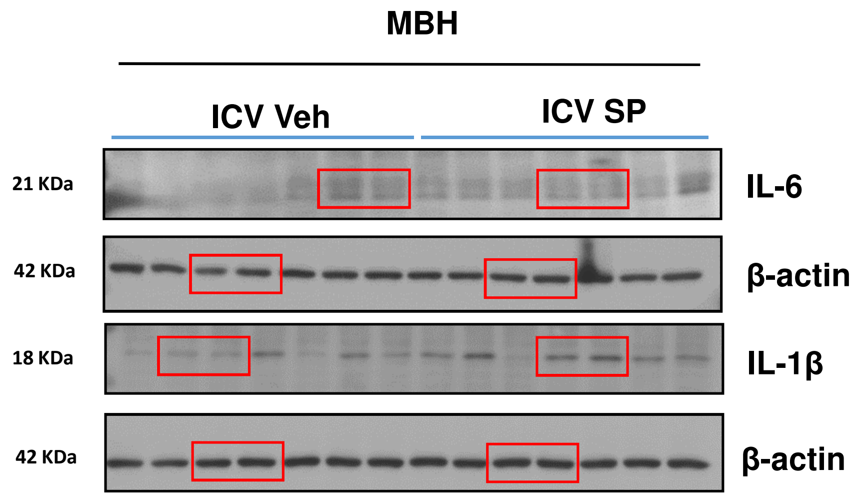

Related to Suppl. Figure S10g

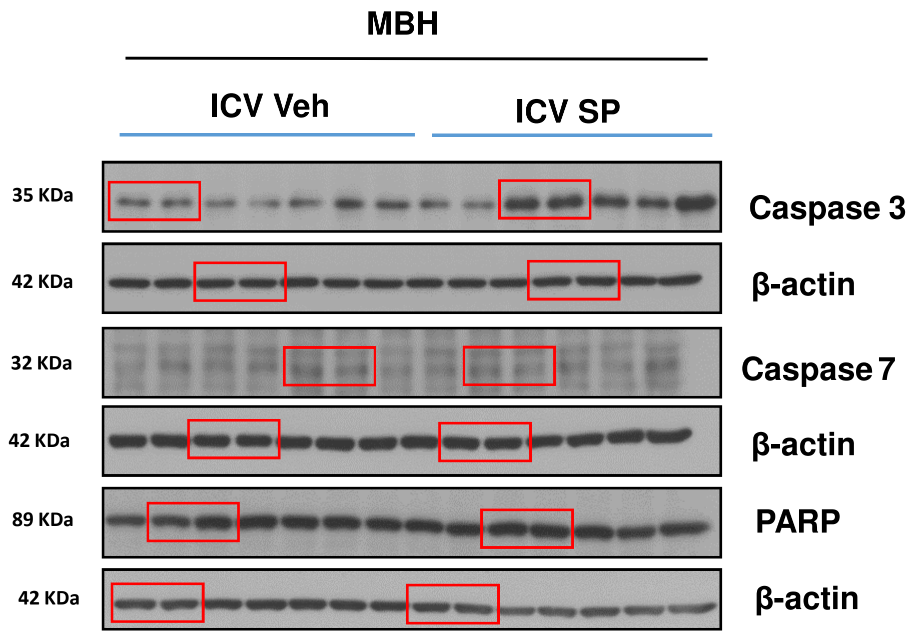

Supplementary figure 16. Appendix figure S10.

Uncropped blots for Suppl. Figure S13

Related to Suppl. Figure S13

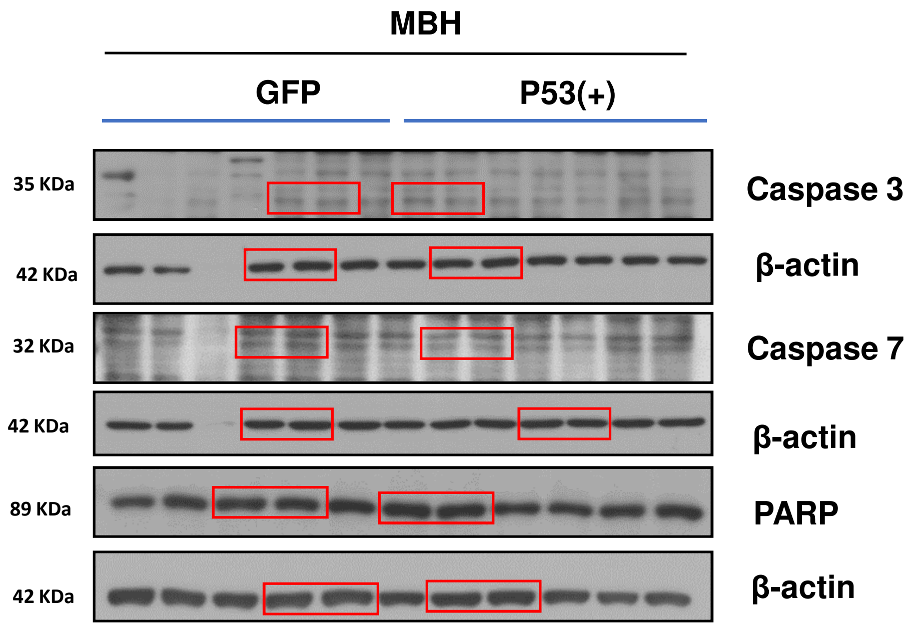

Supplementary figure 16. Appendix figure S13.

Uncropped blots for Suppl Figure S14a

Related to Suppl Figure S14a

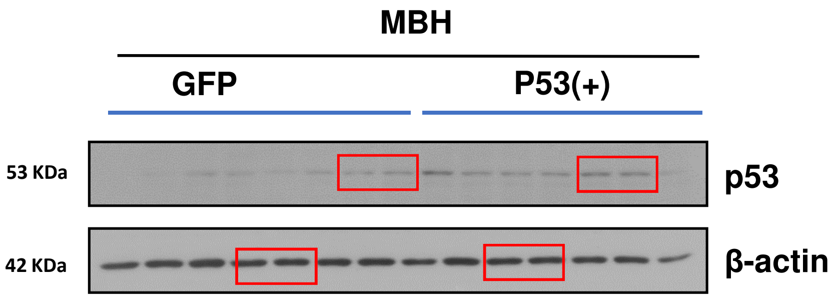

Supplementary figure 16. Appendix figure S14.

Uncropped blots for Suppl. Figure S15h

Related to Suppl. Figure S15h

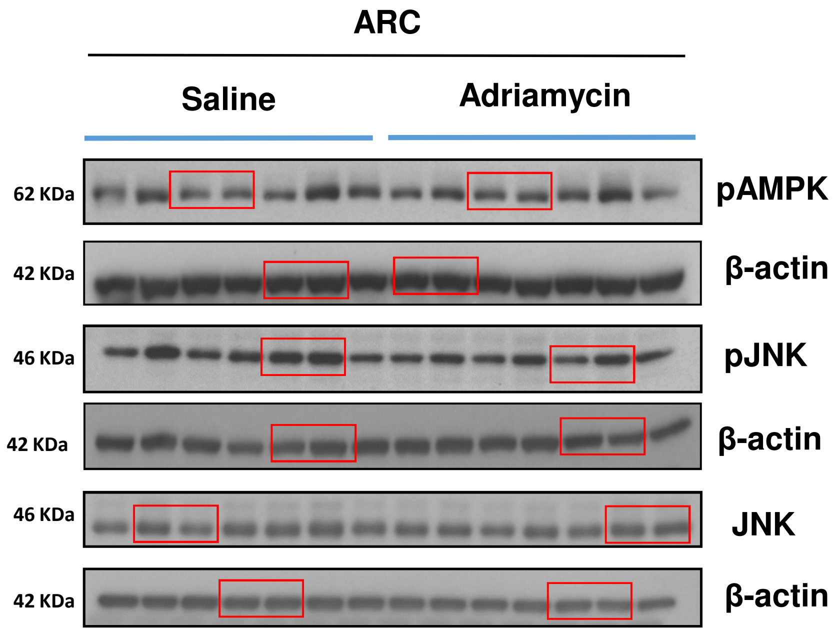

Supplementary figure 16. Appendix figure S15.
